# Genetic variability and genotype by environment interaction of two major cassava processed products in multi-environments

**DOI:** 10.3389/fpls.2022.974795

**Published:** 2022-10-17

**Authors:** Cynthia Idhigu Aghogho, Saviour J. Y. Eleblu, Moshood A. Bakare, Ismail Siraj Kayondo, Isaac Asante, Elizabeth Y. Parkes, Peter Kulakow, Samuel Kwame Offei, Ismail Rabbi

**Affiliations:** ^1^West Africa Centre for Crop Improvement (WACCI), College of Basic and Applied Sciences University of Ghana, Legon, Ghana; ^2^International Institute of Tropical Agriculture (IITA), Ibadan, Nigeria; ^3^Plant Breeding and Genetics Section, School of Integrative Plant Science, College of Agriculture and Life Sciences, Cornell University, Ithaca, NY, United States

**Keywords:** genetic variability, heritability, genotype by environment interaction, gari and fufu conversion rate, cassava processed products breeding

## Abstract

Conversion of cassava (*Manihot esculenta*) roots to processed products such as gari and fufu before consumption is a common practice worldwide by cassava end-user for detoxification, prolonged shelf life or profitability. Fresh root and processed product yield are supposed to be equivalent for each genotype, however, that is not the case. Developing genotypes with high product conversion rate is an important breeding goal in cassava as it drives the adoption rates of new varieties. The objective of this study was to quantify the contribution of genetic and genotype-by-environment interaction (GEI) patterns on cassava root conversion rate to gari and fufu. Sixty-seven advanced breeding genotypes from the International Institute of Tropical Agriculture (IITA) were evaluated across eight environments in Nigeria. Root conversion rate means across trials ranges from 14.72 to 22.76% for gari% and 16.96–24.24% for fufu%. Heritability estimates range from 0.17 to 0.74 for trial bases and 0.71 overall environment for gari% and 0.03–0.65 for trial bases and 0.72 overall environment for fufu% which implies that genetic improvement can be made on these traits. Root conversion rate for both gari and fufu% showed a negative but insignificant correlation with fresh root yield and significant positive correlation to Dry Matter content. For all fitted models, environment and interaction had explained more of the phenotypic variation observed among genotypes for both product conversion rates showing the presence of a strong GEI. Wrickle ecovalence (Wi) stability analysis and Geometric Adaptability index (GAI) identified G40 (TMS14F1285P0006) as part of top 5 genotypes for gari% but no overlapping genotype was identified by both stability analysis for fufu%. This genotypic performance across environments suggests that it is possible to have genotype with dual-purpose for high gari and fufu conversion rate.

## Introduction

Cassava (*Manihot esculenta*) is an affordable carbohydrate source for about 800 million people in Africa, Asia, and Latin America (Montagnac et al., 2009; Dusunceli, 2019). This clonally propagated starchy root crop is considered a food security or insurance crop for smallholder farmers due to its year-round availability, and its ability to grow in marginal environments characterized by water scarcity and poor soils. More than 90% of cassava produced in Africa is used for human food compared with 50% in Asia and 43% in South America, while the remaining 10% is used for animal feed production (Nweke, 2004).

Cassava roots are consumed either fresh or processed into various products. Processing products differ depending on the consumption preference and processing method used. Sweet genotypes with low cyanide content goes through minimal processing such as boiling, roasting or frying while non-sweet genotypes goes through a more rigorous processing which include grating and fermentation before consumption (Lancaster et al., 1982; Nweke, 1994). Various processing techniques are used to add value, extend the shelf life of the products as well as detoxify the roots by removing cyanogenic glucosides (Westby, 2002; Cardoso et al., 2005). Without processing, commercialization of cassava roots for urban markets will be difficult to achieve (Coulibaly et al., 2014). By far, processed products account for the largest proportion of cassava food consumption in Africa absorbing more than 90% of the produced roots (Nweke, 2004).

The processed products derived from cassava roots include flour, starch and various forms of fermented products. In Africa, cassava roots are converted into a diverse set of products, the most important of which are gari and fufu, as well as tapioca, lafun, and attieke (Ezemenari et al., 1998). The processing method which may or may not include fermentation or starch gelatinization (Sanchez et al., 2010) and cell-wall disintegration (Eggleston and Asiedu, 1994) results in products with different attributes related to sensory, pasting and functional properties (Sanni et al., 2003; Onitilo et al., 2007). The end products may be categorized as flour, thick paste, or semolina-like particles (Awoyale et al., 2021).

Also known as cassava semolina or “farinha de mandioca,” gari is the most common processed product of cassava in West Africa due to its long shelf-life and its ready-to-consume characteristic (Oluwafemi and Udeh, 2016). Cassava roots are rendered into gari by peeling, washing, grating, squeezing out water, and roasting on a dry hot surface. The grated mash can either be directly processed or fermented to produce a product with varying degrees of sourness. The resulting product is dry crispy, fine to coarse granular flour. As a result of its pre-gelatinization property, gari can be eaten in the uncooked form, or soaked in cold water like cereal or added to hot water to produce a thick dough called eba and consumed with vegetable sauce (Sanni et al., 1998).

Fufu is the second most common product after gari in Africa (Sanni et al., 1998). It is produced from retted roots after steeping in water for several days to allow for microbial fermentation and tissue disintegration. The raw mash is sieved to remove insoluble fiber (vascular bundles) and cooked directly into doughy meals or dried and milled into flour for longer shelf life (Akingbala et al., 1991). There are different variations to these processing methods depending on region or desired taste (Okpokiri et al., 1985).

Product conversion rate, defined as the percentage of final product relative to a unit of starting fresh roots, is an important factor in determining variety acceptability by growers and processors. Although this has increased productivity on a fresh root weight basis, processing traits related to conversion rates have not been adequately addressed by breeders (Wossen et al., 2017). Processing the same quantity of roots from different varieties may result in different quantities of derived products (on a dry weight basis). This has an important efficiency and economic implication given the fixed cost of product processing such as labor, time, energy and other resources such as transportation. Varieties with high conversion rate are more preferable when everything is held constant (Wossen et al., 2017). Additionally, overall product yield in tons/ha, defined as fresh root yield (t/ha) times the conversion rate of the product is an important overall productivity metric that can vary among varieties. There is also a trade-off between fresh root yield and conversion rate as it influences production and processing efficiency. For example, a variety with high fresh yield but low conversion rate may be less preferable than a moderately yielding variety with high conversion.

Cassava breeding cycle through phenotypic recurrent selection takes up to 8 years before the release of a new variety (Ceballos et al., 2020). Cassava breeding scheme from botanical seed production to varietal release comprises of six selection stages which include progeny testing (F_1_) clonal evaluation stage, preliminary yield stage, advance yield stage, uniform yield stage (regional trial) and one multiplication stages which include farm level testing (Ceballos et al., 2012). Historically cassava breeding has focused on yield improvement, increases nutritional content and pest and disease resistance in developing new varieties which have largely been addressed (Hahn, 1989; Okechukwu and Dixon, 2008). Currently, due to increased cassava cultivation and commercialization worldwide and in Africa, there is increased incentive to breed for varieties that are not only high yielding but also high product conversion rate. The lack of prioritization of these traits in breeding programs can often lead to low adoption of modern varieties (Nweke, 2004; Wossen et al., 2017).

Trait improvement is highly dependent on the availability of information related to genetic component and trait behavior across environments. Genetic variability, heritability, and stability of expression of root conversion rate traits as well as their correlation in cassava is limited, thereby hindering the ability of breeders to improve them through recurrent selection schemes. Heritability, defined as the proportion of phenotypic variation that is attributable to genetic variance, is important in crop improvement as it influences traits evaluation and selection accuracy (Kempthorne, 1970). Breeding selection is relatively easy for traits with large genetic variance and heritability. The influence of the genotype-by-environment interactions (GEI) in the expression of a trait in different environments also need to be considered by breeders in order to identify superior genotypes and of the location that best represents the target environment. Previous studies on conversion rate of processed products have focused on either the effect of different root storage method before processing, different processing methods or genotype harvesting age (Oghenechavwuko et al., 2013; Adegbola et al., 2014; Oyeyinka et al., 2019). Studies associated with genetic variability are limited, descriptive in nature and assessed a limited number of genotypes in one or few environments (Etudaiye et al., 2009; Bassey, 2018). These studies have been unable to estimate the genetic contribution and genotype by environment interaction (GEI) effect as well as relationship between cassava processed product traits and key agronomic variables.

In the present study, we carried out multi-location, multi-year phenotyping trials using advanced breeding lines to: (1) evaluate the genetic variation and heritability of 12 traits related to product conversion rate and overall product yield for two major processed products (gari and fufu); (2) monitor the effect of different environments and to estimate genotype by environment (GEI) interactions; and (3) understand the relationship between processing traits and key agronomic variables such as yield and yield components. We used a collection of advanced breeding clones developed by the International Institute of Tropical Agriculture (IITA), Ibadan, Nigeria.

## Materials and methods

### Plant materials

A total of 62 advanced genotypes and 5 checks from the uniform yield trials (UYT) in the IITA cassava breeding program were used in this study. These genotypes, derived from a second generation of a genomic selection-based population improvement pipeline (Wolfe et al., 2017) were selected based on their performance on fresh root yield, dry matter content, harvest rate, root number. All accessions are resistant to the cassava mosaic disease which is known to negatively influence productivity in cassava (Thresh et al., 1997). The genotypes had white root pulp with moderate to high dry matter percentage. The genotypes were randomly divided into two sets of trials (UYT36setA and UYT36setB). Each set has 31 clonal lines and 5 standard checks in common, making a unique set of clones of 67 genotypes (Supplementary Table 1). The 5 standard checks used for the study include TMS30572 and TMEB419 (most adopted and popular varieties in Nigeria), TMS-IBA000070, a recently released variety, TMS-IBA980581 and TMS-IBA982101 both of which are high yielding and disease resistant varieties.

### Experimental sites and design

Field experiments were conducted across four unique locations in 3 years, the locations used varied from 1 year to another as did the number of trials making a total of eight unique environment with year and location combined (Table 1). These locations represent two major agro-ecological zones in Nigeria (Table 1) which was selected to represent the major regions where gari and fufu products are produced and consumed (Ezedinma et al., 2007). The agroecological variability of the locations include the humid forest characterized by high precipitation and derived savanna with moderate precipitation (Iloeje, 1965). The selected locations represent the major The trials were established in an alpha-lattice design with two replications. Plot dimension was 6 m × 7 m consisting of 42 planted at a spacing of 1 m × 0.8 m^2^ plants between and within rows, respectively. The row and column numbers for each genotype within-trial sets were recorded for spatial trend analysis.

**TABLE 1:** Summary of trial locations, agro-ecological zones, and seasons.

Location	Agro-ecological zone	Year	Latitude	Longitude
Ago-Owu	Derived Savanna	2017, 2018, 2019	7°20′ N	4°16′ E
Ibadan	Derived Savanna	2018, 2019	7°49′ N	3°90′ E
Ikenne	Humid forest	2017, 2019	6°84′ N	3°69′ E
Ubiaja	Humid forest	2019	6°67′N	6°34′ E

### Trial harvesting and yield trait phenotyping

The trials were harvested at maturity, 12 months after planting. To reduce the border effect on genotype agronomic performance, only the net plot consisting of 20 plants were harvested for phenotyping. During harvest, plot-level data on root number (RTNO), root weight (RTWT), and root size (RTSZ) were recorded. Harvest index was recorded as the ratio of root biomass relative to total biomass. Fresh root yield (FYLD) expressed as tons per hectare and was calculated from RTWT adjusted by plant spacing of 0.8 m^2^. Dry root yield (DYLD) was derived as a product of Dry matter (DM) content and FYLD. RTSZ was recorded categorically as 3 (small), 5 (average), and 7 (large) as recommended by Fukuda et al. (2010). All data was captured using FieldBook app (Rife and Poland, 2014) and traits recorded using established ontologies from www.cassavabase.org.

### Product processing

Marketable roots from each plot were selected for dry matter estimation and processing into gari and fufu products. To access the root dry matter content, 6–8 roots were randomly sampled, peeled, and grated after removing proximal and distal ends to reduce fibrous material. After thorough mixing, 100 g samples of the root grates were oven-dried at 95°C for 48 h until constant weight and the dry matter was expressed as a percentage of fresh weight. Care was taken to ensure rotted or damaged roots are not included in the sampling.

Each product was processed from 20 kg of roots but 10 kg was used when sufficient quantity was not available. The roots from each plot were packed in separate pre-labeled bags and transported to centralized facilities to ensure processing is done the same day and reduce post-harvest physiological deterioration.

### Gari production

Gari processing was carried out as described in Ukhun (1989). Peeled roots were washed, and grated into fine particles. The grated mash was transferred into woven polypropylene sacks and allowed to undergo spontaneous fermentation for 48 h. Water was pressed out of each sample using a hydraulic method to eliminate about 60 percent of the remaining water. The semi-dried cakes were then sieved and toasted in a hot stainless-steel frying tray to form gelatinized, dry and crispy granules. The temperature of the copper tray before frying ranges between 143.67°C and 148.87°C and the final temperature of the fried product is 88.01°C–90.93°C. Finally, the gari product was allowed to cool to room temperature and stored in barcoded nylon bags after recording the product weight in kg. Conversion rate was calculated as a percentage of starting fresh root as follows:


Gari%=FinalgariweightStartingrootweightX 100

### Dried fufu processing

Fufu processing followed the method of Achi and Akomas (2006). This method produces either odorless fufu paste or dried to produce a flour-like product. In this study, we generated dry fufu product for estimating conversion rates. The dry fufu is suitable for long term storage.

After peeling, roots were cut into small chunks and soaked in individual plastic buckets using 40 l of water for 4 days to undergo lactic acid fermentation until the roots are softened. After softening, the starchy pulp was separated from insoluble fiber using a 0.3 cm pore size sieve over a clean bucket. The pulp filtrate was washed twice with 20 l of clean water and allowed to sediment for 4 h until the water clears. The water was carefully decanted and the product transferred to a cotton bag followed by straining of the remaining water. Finally, the product was spread on clean flat stainless-steel trays and oven-dried at 60°C for 48 h to a constant weight. The resulting odorless fufu was allowed to cool to room temperature and stored in barcoded nylon bags after recording the product weight in kg.


Fufu%=driedfufuweightStartingrootweightX 100

Processing losses in terms of peel weight and dried insoluble fiber removed from fufu mash were also recorded. Conversion rate is a function of % moisture content and peel waste, which can be up to 35% of root proportion (Omosuli et al., 2017). For fufu, processing losses also includes insoluble fiber that is removed after sieving and placed in a 60°C oven for a period of 48 h before weighing in kg. Peel loss and fiber content were converted to percentage of initial weight of root used for processing into product as described below:


Peelloss%=PeelweightStartingrootweightX 100

Fibrecontent%=DriedfibreweightStartingrootweightX 100

### Statistical analyses

Descriptive statistics per environment and trait were generated and visualized in R (R Core, 2020). The distribution of observed traits using BLUPs was visualized with violin plot with boxplot superimposed and stacked plots across trials using the ggplot2 package (Wickham, 2016) in R (R Core, 2020).

In order to estimate variance components of traits, a single trait linear mixed model was fitted using the ASReml-R package version 4.1 (Butler et al., 2017), considering the genotype, replication, environment, and genotype by environment as random effects while sets, rows and columns of each trial as fixed effects of accounting for trial design-related variables.

We fitted a model as shown in Eq. [1]:


y=Xβ+Zu+e[1]

With $u∼N\left(0,I\sigma_{u}^{2}\right)$ and $\text{e}∼N\left(0,I\sigma_{e}^{2}\right)$, where **y** is the response vector of a trait for a given location, β is the vector of fixed effects with the design matrix **X** (relating observations to fixed effects which include grand mean, row number nested within set and column number nested within set); **u** is the vector of random genetic effects with the design matrix **Z** (relating trait values to genotype, environment, replication nested within environment and GEI) and e is the residual. Test of significance of variance components was done using *z*-test as done in ASReml-R package version 4.1 (Butler et al., 2017).

The BLUP represents an estimate of each individual’s total genetic value across environments for the genotype effect. For the sake of correlation analysis, it is important to estimate a single value that encapsulates all the information available on the individual; we estimated the de-regressed BLUPs (D-RBLUPS) by dividing by their reliability $deregressedBLUP=\frac{BLUP}{\left(1-\frac{PEV}{\sigma_{i}^{2}}\right)}$ (Garrick et al., 2009) where PEV is the predicted error variance of the BLUP and $\sigma_{i}^{2}$ is the clonal variance component.

Correlation analysis of the traits using the D-RBLUPS estimates was determined using the *corrr* package and visualize using *ggcorrplot* in core R version 4.1.1 (R Core, 2020).

Broad-sense heritability was estimated using two methods. First, the standard method (H2_standard) based on error plot variance across all environments was derived from variance components estimated as $H^{2}=\frac{\sigma_{g}^{2}}{\sigma_{g}^{2}+\left(\frac{\sigma_{ge}^{2}}{e}\right)+\left(\frac{\sigma_{\varepsilon }^{2}}{er}\right)}$ where $\sigma_{g}^{2}$ refers to the variance of genotype, $\sigma_{ge}^{2}$ is GEI variance, $\sigma_{\varepsilon }^{2}$ is the environmental variance, *e* is the number of environments, *r* is the number of replicates of genotypes per environment, and other terms were described above. The second broad-sense heritability (H_Cullis) proposed by Cullis et al. (1996) was estimated using genotype standard error calculated as; $H_{cullis}^{2}=1-\frac{{\bar{v}}_{△}^{BLUP}}{2\sigma_{g}^{2}}$ where $\sigma_{g}^{2}$ refers to genetic variance, ${\bar{v}}_{△}^{BLUP}$ to the average standard error of the genotypic BLUPs.

To carry out GEI, we generated table of genotype means by fitting a mixed model with genotype as fixed effect as shown in Eq. [2]:


y=Xβ+Zu+e[2]

With $u∼N\left(0,I\sigma_{u}^{2}\right)$ and $\text{e}∼N\left(0,I\sigma_{e}^{2}\right)$, where **y** is the response vector of a trait for a given location, β is the vector of fixed effects with the design matrix **X** (relating observations to fixed effects which include grand mean and genotype); **u** is the vector of random genetic effects with the design matrix **Z** (relating trait values to environment, row number nested within set and column number nested within set) and e is the residual.

The resulting table of Best Linear Unbiased Estimates (BLUEs) was used to model the GEI using three approaches shown in Table 2; Malosetti et al. (2013) using the statgenGxE package version 1.0 (van Rossum et al., 2021) in R version 4.1.1 (R Core, 2020).

**TABLE 2:** Description of models and references.

Model	Formular	Variables	References
FW	*y*_*ij*_=μ+*G*_*i*_+*E*_*j*_+*b*_*i*_*E*_*j*_+*e*_*ij*_	*y*_*ij*_ is the mean yield of genotype *i* in environment *j* μ is the grand mean *G_i* is the genotypic effect; *E*_*j*_is the environment effect; *b*_*i*_*E*_*j*_is a sensitivity parameters; *e*_*ij*_ is the residual.	Malosetti et al., 2013
AMMI	$y_{ij}=\mu +g_{i}+e_{j}+\sum_{k=1}^{K}\lambda_{k}a_{ik}\gamma_{ij}+\varepsilon_{ij}$	*y*_*ij*_ is the mean yield of genotype *i* in environment *j*; μ is s the grand mean. *g_i* is the genotype fixed effect of j*^th^* environment. The GEI component is decomposed into K multiplicative terms (k = 1, 2, …, K), each multiplicative term is a product of k*^th^* eigenvalue (k); genotypic score (ik); and environmental scores (jk); and ij is the residual	Gauch, 1992
GGE	$y_{ij}=\mu +e_{j}+\sum_{k=1}^{K}\lambda_{k}a_{ik}\gamma_{ij}+\varepsilon_{ij}$	Terms are similar to AMMI model but without *g_i* which is the genotype fixed effect of j*^th^* environment.	Yan et al., 2000

The first approach was suggested by Finlay and Wilkinson (1963) (FW), a regression analysis which has been widely used to describe stability and GEI in various cultivars (Mulusew et al., 2014; Swanckaert et al., 2020). The Finlay-Wilkinson regression model estimates the heterogeneity of slopes and sensitivity of a genotype by regressing mean phenotypic performance of individual genotypes on an environmental index using within-line ordinary least squares (OLS) regression (Lian and de los Campos, 2016). However, FW linear regression is not sufficient to fully explain the genotype phenotypic stability.

Before fitting the FW model (Table 2), trait values were scaled to mean of zero and a standard deviation of 1, following the equation below as: $\mathrm{adjusted}\mathrm{phenotype}\mathrm{mean}\mathrm{scaled}=\frac{\left[y_{ij}-mean\left(Y\right)\right]}{sd\left(Y\right)}$ where *y*_*ij*_ is the adjusted phenotypic mean value of i*^th^* genotype in j*^th^* environment and sd (Y) is the standard deviation of the overall mean of the adjusted phenotypic response of all clones in all environments. The scaling allowed the comparison of MSE and sensitivity values across traits that are originally on different scales and units measurement (Falcon et al., 2020).

The second and third approaches are Fixed-effect linear-bilinear models Additive Main-effects and Multiplicative Interaction (AMMI) and Genotype Main Effect plus Genotype-Environment Interaction (GGE). Both approaches depend on analysis of variance (ANOVA) for estimating genotype and environment main effect, principal component analysis (PCA) for decomposing GEI structure into Interactive Principal Component Axes (IPCAs) and biplot for graphical presentations. The AMMI model can be further used to delineate the testing environments into mega environments using principal component axes scores. AMMI gives a suitable approach in separating genotypic effect from genotype by environment effect with cultivar ranking in mega-environment (Hagos and Abay, 2013) while GGE is suitable for grouping sites and cultivars without cultivar rank change (Yan and Hunt, 2001). Despite their different approaches, both models complement each other in order to strengthen decision making thereby permitting increased reliability in the selection of superior cultivars and test environments.

### Stability of genotype performance across environments

We assessed stability of genotypes for traits observed using both static and dynamic stability measures. Static stability was measured using Wrickle ecovalence (Wi) as proposed by Wricke (1962) and described $W_{i}^{2}=\sum {\left(X_{ij}-\bar{X}i.-\bar{X.}j+\bar{X}..\right)}^{2}$ where *X*_*ij*_ is the observed trait respond (average across replication), $\bar{X}i.$ correspond to the mean yield of genotype *i*, $\bar{X}$.j is the mean yield of the environment *j* and $\bar{X}$ is the grand means.

According to the ranking of genotypes by Wi, stable genotype has lower Wi preferably close to 0. These are genotypes that have smaller deviation from the environmental mean. Likewise, genotypes with high Wi indicates instability in genotype performance across the environments and a large contribution of the genotype to the GEI.

Geometric adaptability index (GAI) is a measure of the adaptability of a genotype and is classified as a dynamic concept of stability (Mohammadi and Amri, 2008) and described as: $\text{GAI}=\sqrt[{E}]{\bar{X}1+\bar{X}2+\ldots +\bar{X}l.}$ Where $\bar{X}1$, $\bar{X}2$, and $\bar{X}l$ are the mean yields of the first, second and *i*th genotypes across environments and E is the number of environments. According to the ranking of genotypes by GAI, genotypes with the high GAI (low ranks) are desirable (Mohammadi and Amri, 2008; Pourdad, 2011), Wi and GAI was estimated using metan package version 1.15 (Olivoto and Lúcio, 2020) in R version 4.1.1 (R Core, 2020).

## Results

### Analysis of variation for cassava gari, fufu, and related yield traits evaluated in multiple environments

**FIGURE 1:**
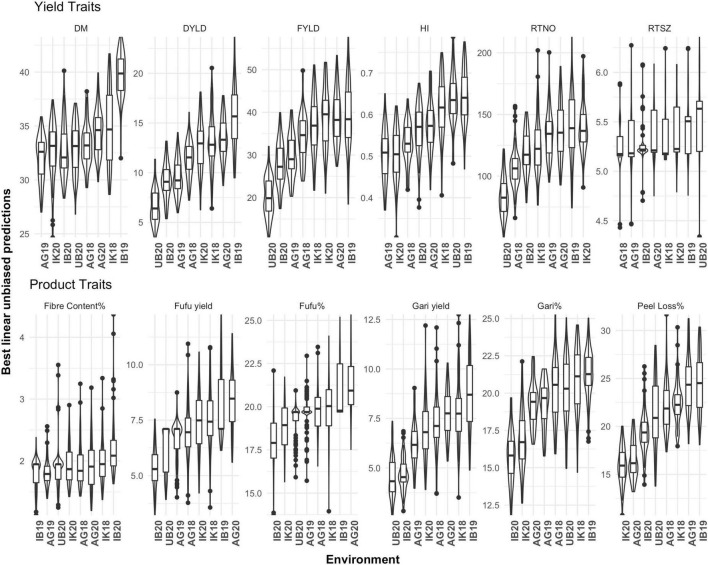
Phenotypic variation of 12 yield and product traits of cassava across eight environments. IB19, Ibadan 2019; IK18, Ikenne 2018; AG19, AgoOwo 2019; UB20, Ubiaja 2019; IK20, Ikenne 2020; Env, Environment; DM, Dry matter; DYLD, Dry root yield; FYLD, Fresh root yield; HI, Harvest rate; RTNO, Root number; RTSZ, Root size.

Across environments and genotypes, a considerable range of phenotypic values was observed among genotypes and trials as shown in the distribution of estimated BLUPs (Figure 1). We observed differences between trials for FYLD which ranged from low performing environment (UB18, 20.18 t/ha) to high performing environment (IB19, 39.22 t/ha) with an average of 33.03 t/ha. In the study population we also observed an average of 36.94% for DM with genotypes ranging from 24.67 to 43.26 across environments. There was also variation in DM among trials used in this study ranging from lowest average DM content in AG19 (31.99) and highest IB19 (32.66). An average of 19.23% was observed for gari% with a variation between genotypes ranging from 11.82 to 25.27% across the eight environments used in this study. Between trials UB20 (20.24%) had the lowest average gari% while IB19 (21.29%) had the highest average gari%. Fufu% also appears to have an average phenotypic variation of 19.68% across the eight environments with genotypes ranging from 13.72 to 25.34%. We also observed trial variation for fufu% with UB20 (19.22%) having the lowest average and AG20 (21.25%) having the highest average fufu%. The results from phenotypic variability for fufu% is not far from what we observed in gari%. In the study population the average peel loss% was 20.80% but can range from as low as 10.75% to as much as 31.97% for some genotypes. Among the trials we had the lowest average peel loss% recorded in IK20 (15.93%) and highest was observed in IB19 (25.58%).

**FIGURE 2:**
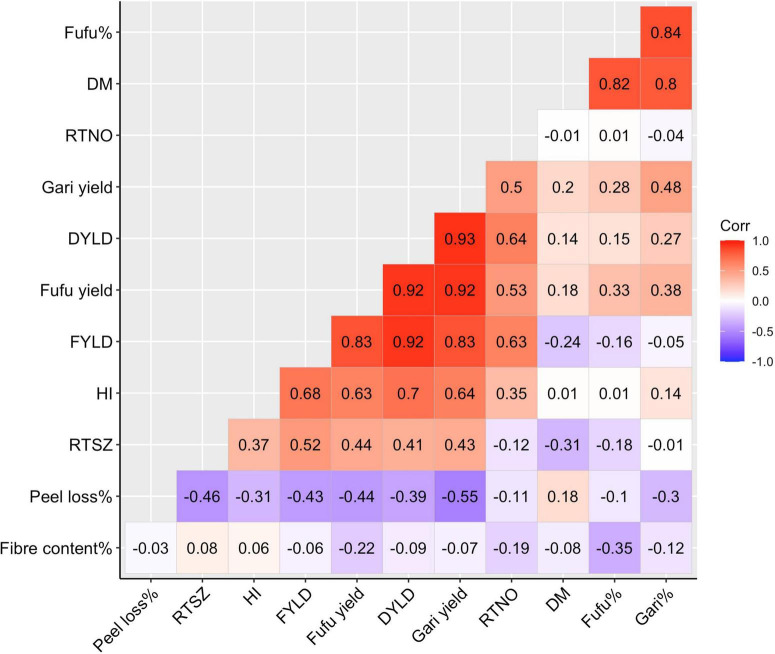
Correlation plot for processed products traits, and yield and root morphological traits using deregressed BLUPS form 8 environments. DM, Drymatter; RTNO, Root number; DYLD, Dry root yield; FYLD, Fresh root yield; HI, Harvest index; RTSZ, Root size.

Relationship between all studied traits is represented using a correlation matrix (Figure 2). There was a positive significant (*P* < 0.05) correlation between DM and gari% (*r* = 0.80) and between DM and fufu% (*r* = 0.82) as well as between gari and fufu% (*r* = 0.84, *P* < 0.05). Interestingly, there appears to be no relationship between yield and the conversion rates as well as DM. We could see from the correlation matrix that DYLD, FYLD, gari yield, and fufu yield (yield traits) were highly correlated among each other. The yield traits correlation is not surprising because all yield traits were derivatives of RTWT. We observed a negative correlation between Fiber content% and fufu% (*r* = –0.35, *P* < 0.05). The fiber content% of a genotype is highly dependent on the ability of the genotype to soften during the fermentation stage in processing. Genotypes with high softening ability will produce less fiber which will be desirable for production of high fufu%. The correlation matrix also showed a negative correlation of peel loss% and gari% (*r* = –0.30, *P* < 0.05) and FYLD (*r* = –0.43, *P* < 0.05).

**FIGURE 3:**
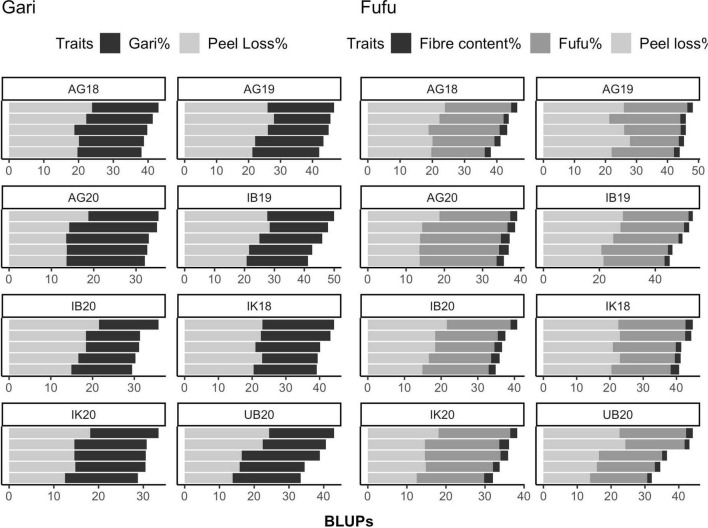
Relationship between gari and fufu % and processing loss traits for eight environments. IB19, Ibadan 2019; IK18, Ikenne 2018; AG19, AgoOwo 2019; UB20, Ubiaja 2019; IK20, Ikenne 2020.

To further understand the relationship between both conversion rates (gari% and fufu%) and processing losses (peel loss% and fiber content%) can be visualized in Figure 3, we observed a 1:1 ratio in average values among genotypes for gari% (19.23%), fufu% (19.68%), and peel loss% (20.87%). This ratio means that the overall performance of a genotype is dependent on peel loss%. We recorded fiber content% which is related to fufu processing to be about 4.5% across trials. Among both processing losses, peel loss% has the highest contribution compared to fiber content%.

**FIGURE 4:**
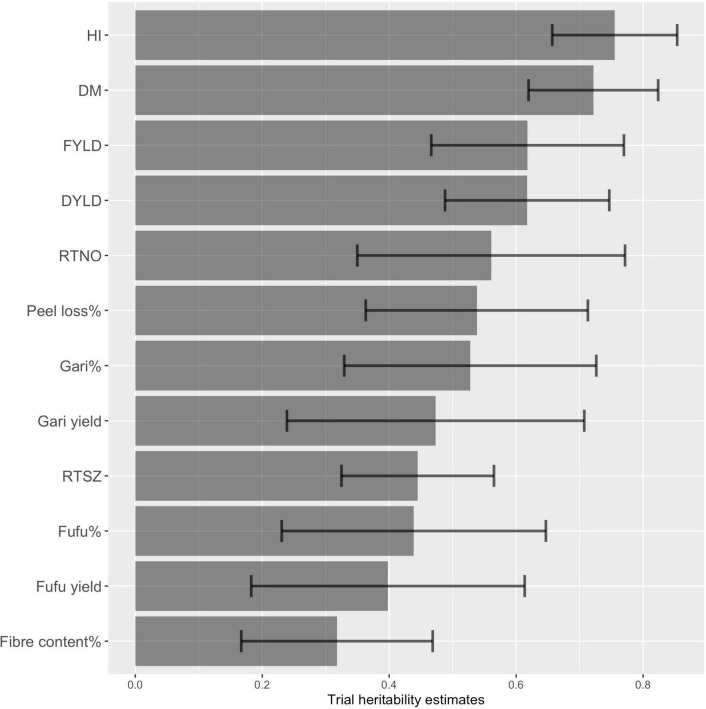
Broad-sense heritability estimated for 12 processed product and yield traits based on H2_Cullis method with error bars (standard deviation). DM, Drymatter, RTNO, Root number, DYLD, Dry root yield; FYLD, Fresh root yield; HI, Harvest index; RTSZ, Root size.

Because of the observed variation among trials, heritability estimates were computed using the mean of the genotypes within each trial and square of the standard error of the genetic estimates (Figure 4 and Supplementary Table 2). We computed both H2_standard and H2_Cullis estimates were comparable for all 12 traits, ranging from 0.31 to 0.75 and 0.6–0.8, respectively. Gari% and fufu% had a higher H2_standard (>0.70) compared to H2_Cullis (>0.40). We observed a within trial heritability estimated using H2_Cullis method (Figure 4) for gari% ranging from 0.18 to 0.74 with standard deviation (error bars) of 0.19 and fufu% ranges from 0.03 to 0.65 with standard deviation of 0.21. Some traits such as FYLD, DYLD gari and fufu yield had heritability estimates higher than 0.60 using the H2_standard method and higher than 0.40 using the H2_Cullis method. The lower H2_Cullis heritability estimates observed for these traits is due to inter-trials variations. The most relevant processing traits (peel loss%) had a heritability estimate of 0.54 using the H2_Cullis method and 0.81 using the H2_standard. The moderate to high levels of heritability observed for gari and fufu% indicates a higher proportion of genetic to total phenotypic variability which is suitable for genetic improvement of these traits through recurrent selection.

**FIGURE 5:**
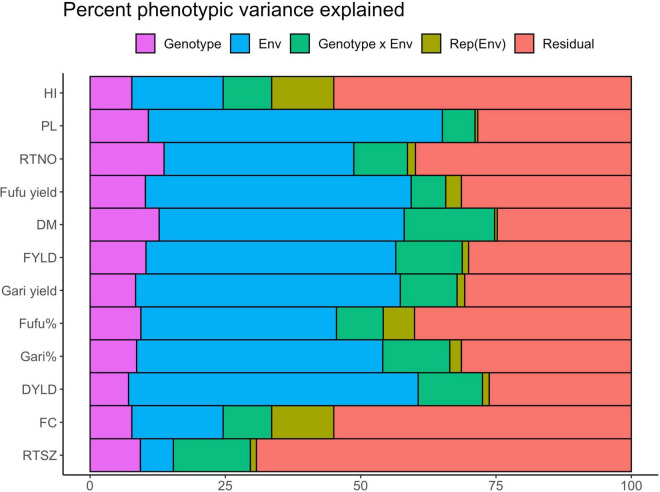
Percent of phenotypic variance explained by each fixed model analysis of variance model for 12 processed product and yield related traits. Env, Environment; rep, Replication; DM, Drymatter; RTNO, Root number; DYLD, Dry root yield; FYLD, Fresh root yield; HI, Harvest index, RTSZ, Root size.

**FIGURE 6:**
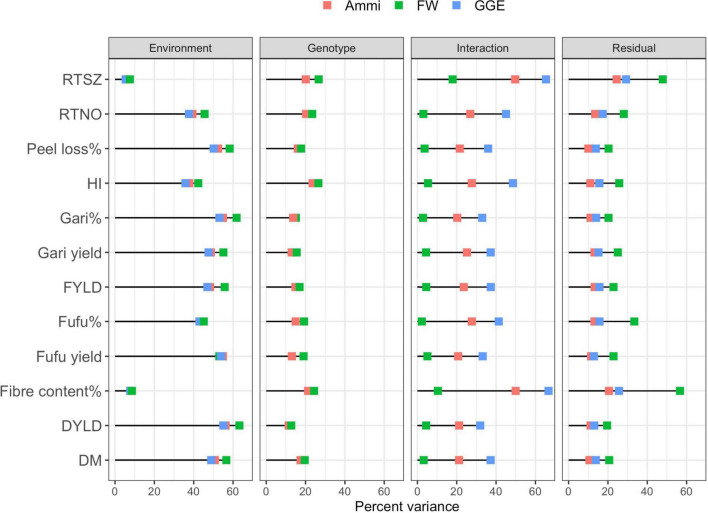
Partitioning of total sum of square (TSS) based on total sum of squares captured by each factor from fitting additive main effect and multiplicative interaction (AMMI) (represented by the orange square), Finlay Wilkinson (FW) (represented by green square), and genotype and genotype by environment (GGE) (represented by blue square) models on 67 elite cassava genotypes evaluated in 8 environments. DM, Drymatter; RTNO, Root number; DYLD, Dry root yield; FYLD, Fresh root yield; HI, Harvest index; RTSZ, Root size.

Results from the linear mixed model fitted to explain the contribution of genotype, environment, GEI, replication within environment and residual to phenotypic variation is presented in Figure 5 and sorted according to the descending H2_standard heritability estimates. We observed that environment had a significant effect on all traits (P < 0.01) ranging from 6.10 in RTSZ to 54.33% in peel loss% and explained the largest percentage of variation for most traits except for RTSZ (6.10%), fiber content% (16.87%) and HI (16.87%). Gari and fufu% had 45.48 and 36.14% of phenotypic variation explained by the environment, respectively, which was higher than what was explained by genotype effect. About 8.59 and 9.37% of phenotypic variation was explained for gari and fufu%, respectively, by the genotype term. The genotypic effect was also found to be significant (*P* < 0.001) for all traits and explained between 7.09% (DYLD) and 12.76% (DM). The significant genotypic terms suggest that these traits are influenced by genes and not only the environment. Change in relative performance of genotypes across environments is explained by the GEI term. There was a significant (P < 0.001) contribution of genotype x Env term to phenotypic variation observed among genotypes for most of trait in our study population except for fiber content% and explained between 6.03% (peel loss%) to 16.71% (DM) of phenotypic variation. Gari and fufu% phenotypic variance explained by GEI as 12.39 and 8.65%, respectively, which is close to what was explained by the genotype term. The replication nested within the environment which explains the experimental design effect captured the least percentage variation (0.50–11.48%) and was insignificant (Supplementary Table 3). The second-largest source of variation after the environment team accounting for up to 69.27% for RTSZ (Figure 6) is the residual term. This means there are some unexplained variations that could not be explained by the other terms in the model.

## Genotype by environment interaction

### Finlay-Wilkinson regression

The genotypic and environmental main effects of the Finlay-Wilkinson (FW) model were highly significant (*P* < 0.001) for all observed traits (Supplementary Table 4). However, the interaction effect was not significant for all traits except DYLD and RTSZ. According to the partitioning of the total sums of squares (TSS) (Figure 6), the environment term had the largest contribution for most traits ranging from 42.34% (HI) to 61.81% (gari yield). Of all the terms in the FW model, the interaction term has the least contribution to the TSS while the error term contributed almost twice the percentage contributed by genotype term.

Significant differences in regression slope (sensitivity) among genotypes on the environmental mean was found for all traits except dry matter content (Supplementary Table 5). In other words, there was variation in genotypic response for all traits but not dry matter with respect to changes in environment mean. Genotype and trait sensitivity to GEI was explained using the variance of the slopes and the variance of the mean square deviation, respectively, which was extracted from the FW regression analysis.

The genotypic sensitivity values were ranked from the most stable (low sensitivity values) to the least stable for each trait (high sensitivity values) for each trait (Supplementary Table 6). Using the FW we identified G38, G32, G24, G3, and G17 as top five stable genotypes for gari% and a different sets of genotypes as stable for fufu% which include G19, G35, G32, G39, and G5 except for G32.

Traits-specific environmental stability can be approximated using slope of the regression. Traits with narrow tolerance to distribution (higher slope) are more sensitive to the effect of environmental stress. The slope variance observed among traits varied from 0.04 (gari%) to 23.14 (fiber content%) with their corresponding slope median values varied from 1.01 and 0.95, respectively (Supplementary Table 4). The higher variance observed for fiber content% from FW regression analysis makes it a rather difficult trait to phenotype because of the large variation in some environments in comparison with others. Fufu% had a slope variance of 0.16 and median of 1.06. Peel loss had the lowest median MS deviation of all traits (median = 0.21) and the variance of MSE (variance = 0.03). Root size and Fiber content had the highest MS deviation. Among all traits observed, genotypes used as checks did not rank first as stable genotypes.

### Additive main effect and multiplicative interaction

The Additive main effect and multiplicative interaction *(AMMI*) analysis revealed significant variation in the main effects of genotype, environment and their interactions (GEI) (P < 0.001) for all observed traits (Supplementary Table 4). The partition of total sum of squares (TSS) (Figure 6) showed that the environment main effect accounted for the highest amount of variation varying from 5.72% (RTSZ) to 54.85% (gari%). Traits with high TSS explained by environment indicate the existence of a group of environments sharing the same genotype(s) as best performing with large differences among environmental means (Yan and Rajcan, 2002). In the study population, the genotype contribution to TSS varied between 11.39% (DYLD) and 23.55% (HI). It is interesting to find out that DM (17.44%) had an almost 1:1 ratio of TSS explained by genotypes for gari% (13.75%), peel loss% (16.03%), and fufu% (14.97%). The ratio observed between traits points out the presence of genetic control for gari and fufu% that can be exploited for traits improvement through recurrent selection in multiple environments. We further decomposed the variation due to GEI for gari and fufu% using the first and second IPCAs and found out that both IPCAs accounted for 20.27 and 27.65% the TSS. For all traits measured in this study, the first and second IPCAs accounted for between 20.27% (gari%) and 49.96% (fiber content%) of the TSS due to GEI. Residual term explained between 10.01% (peel loss%) and 24.43% (RTSZ) of the TSS. We observed an equivalent proportion of TSS explained by residual and genotype terms for all traits except for HI which had a lower % explained by the residual term.

### Genotype and genotype by environment

The GGE analysis of variance for 67 genotypes revealed a significant main effect of environment and combined genotype and GEI effect (*P* < 0.001) for the observed traits (Supplementary Table 4). To discern the contribution of different terms fitted in the model we partition the environment and interaction (first and second IPCAs) sum of squares as percentage of the total sum of squares. After partitioning the TSS we observed that between 5.38% (RTSZ) and 55.06% (fufu yield) of TSS was explained by the environment term and 31.93% (DYLD) to 66.68% (fiber content%) attributed to the interaction term. For gari and fufu%, TSS explained by the environment term was 53.10% which was larger than what the interaction term explained (Figure 6). However, for fufu% both the environment (42.92%), and interaction (41.46%) terms explained a 1:1 ratio of TSS.

Comparing the statistical models used in dissecting the GEI effects of traits observed in this study, we observed that most of the phenotypic variation seen in most traits were explained by the environment term. We also noticed that the contribution of interaction varies greatly among all statistical methods, GGE and FW had the highest and lowest contribution of the Interaction terms to traits phenotypic variation, respectively. This may be due to the removal of the genotype term when fitting GGE model and the sensitivity parameter in the FW model. Of all the variance terms measured, the environment had the highest contribution to traits expression, followed by interaction and residual before genotype. However, a single conclusion that can be drawn from the output indicates that the genotypes present different behavior for different traits in all environments used for this study and the environment was the primary source of variation. The significant interaction terms can affect the attainment of genetic advance from phenotypic selection due to differential response of genotypes under the target test environments.

**FIGURE 7:**
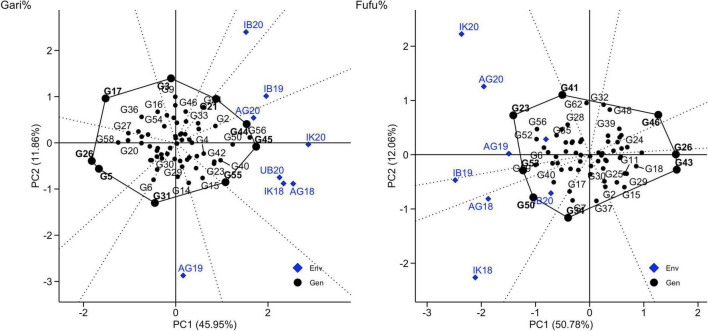
Vector views of PC2 are plotted against PC1 for environment relations, winning genotypes, and environment correlations.

GGE biplot which allows the visualization of genotype, environment and interaction based on symmetric scaling was used to understand the type of GEI in this study for observed traits (Figure 7, Supplementary Figures 1–10). The GGE biplot explained about 57.81 and 61.05%, of the total G + GE interactions (PC1 + PC2) for gari% and fufu%, respectively. We recognized a crossover type of GEI for gari and fufu %, which was indicated by the biplot principal component scores (PC1 and PC2) having both negative and positive values. Additionally, the polygon vertices of the biplots are markers for highly projected genotypes indicating performance in environments in the polygon vector. G45, G44, and G55 had the highest gari% above the environmental means in IK18, AG18, UB20, IK20, and AG20 while G56 and G21 were projected as the best performing genotypes in terms of fufu% in IB19, AG18, UB20, IK20, and AG20. Though the projected best performing genotypes for gari and fufu% in all environments were different, the environment seems to be correlated as indicated by the distance of the environment from origin and angle with other environments. The environmental correlation implies that one environment can represent another in screening for gari and fufu% (Solonechnyi et al., 2015; Temesgen et al., 2015).

### Stability of genotype performance across environments

#### Wrickle’s ecovalence

According to Wrickle (1962) genotypes with low ecovalence index have smaller fluctuation in performance across environments and are desirable because they are more stable. These genotypes, G13, TMS980581, G40, G47, and G12 had the lowest Wi less than 0.48 for gari while G50, G52, G14 G8, and G18 had the lowest Wi less than 0.17 for fufu% (Supplementary Table 7). These genotype had limited differential response to the different environments used in this study. Among the genotypes that showed low Wi for gari%, G40, G47, and G12 while G50, G14, and G18 for fufu% were above the population mean and can be recommended for wide adaptation (Seife and Tena, 2020). Unstable genotypes for gari% include G3, G39, G31, G6, and G21 while for fufu% include TMS982101, G26, G56, G21, and G31.

#### Geometric adaptability index

GAI is used to evaluate genotypes stability based on the geometric mean of genotypes across environments; thus, genotypes with high GAI and low GAI rank are desired (Mohammadi and Amri, 2008; Pourdad, 2011). Results from the GAI (Supplementary Table 7), top 5 genotypes with the lowest ranks for gari% includes G56, G50, G40, G23, and G55 while top 5 genotypes for the lowest ranks for fufu% include G56, G24, G21,TMEB419, and G23. These genotypes had relatively high gari and fufu% above the population mean of 20.10 and 20.93%, respectively. Genotypes with the highest ranks for gari% G45, G44, G49, G35, and G37 while G52, G8, G29, G27, and G30 had the highest rank for fufu%.

## Discussion

Accessing genetic variability and heritability, GEI pattern and relationship between processing traits and key agronomic variables is an important step in trait genetic improvement through recurrent selection schemes. In this study we assessed 12 traits including processed product and key agronomic variable performance of 62 breeding lines compared with five checks, in multi-environment field trials. The environments used in this study include major areas where cassava is converted into processed products and falls under two major agro-ecological zones in Nigeria. The study population revealed large phenotypic variation among genotypes in regards to all traits and between trials (Figure 1). The range of genetic variability observed among genotypes in this study for gari% was in range with what (Ibe and Ezedinma, 1981; Achinewhu et al., 1998; Amoah et al., 2010) reported for five genotypes. However, the range of genetic variability measured for gari% (11.82–25.27%) in this study was lower than what was reported for the two traits harvested at different age which ranged from 22.00 to 52.00% (Adegbola et al., 2014). The range of genetic variability observed among genotypes in this study for fufu % was similar with what (Awoyale et al., 2020) reported for ten improved varieties to assess their suitability for fufu production. Therefore, genetic improvement can be done through recurrent selection for conversion rate of processed products.

Selection efficiency can be improved with a proper understanding of the relationships between traits. The correlation analysis done between processing traits and key agronomic variables reveal a strong positive correlations between gari and fufu% with dry matter content which connotes that dry matter content could be used as a proxy selection parameter to evaluate genotypes which agrees with the results of Laya et al. (2018). The findings in this study supports previous suggestions from Apea-Bah et al. (2009) and Teeken et al. (2021) that an increase in dry matter content would increase conversion rates of processed products. The correlation observed between gari and fufu% with dry matter content is not surprising because the final stage of root conversion into gari% (Frying) and fufu% (oven drying) aim to remove moisture for maximum increase in shelf life of products.

Contrary to expectations, we did not find any relationship between FYLD and gari and fufu% meaning that genotypes with high FYLD do not translate to high root conversion rate for both processed products in the population used for this study. This finding is in line with what Ibe and Ezedinma (1981) reported for 12 cassava cultivars. However, FYLD is still an essential trait for cassava breeding as it is the first attraction to farmers to a variety before processed products’ potential. Another interesting relationship found in this study was the 1:1 ratio between gari% and fufu% with peel loss% (Figure 4) which was similar to what Hahn (1989) reported for 12 varieties. Furthermore, RTSZ may not be directly related to gari and fufu% but it is a strong determinant for peel loss% making root size an essential component for selection when breeding for high Gari% and Fufu%.

Both methods of heritability estimates (H2_Standard and H2_Cullis) for traits revealed high to moderate estimates which emphasizes genetic contribution to these traits in the population used in this study. However, we observed low heritability estimates from the H2_Cullis methods for some trials and should not be misunderstood as a consequence of no genetics contributing to the expression of the trait but may be due to the effect of either environment or processing. There is room for improvement on achieving increased heritability estimates for these traits by further optimizing processing methods or high throughput phenotyping methods.

According to the linear mixed model, FW, AMMI and GGE analysis of variance, a significant genotype effect was observed for the traits measured; this indicated that genotypes were significantly different, hence genetic improvement could be achieved through hybridization and recurrent selection. Furthermore, we observe a change in genotypic performance in different trials which was confirmed by the largest percentage of total sums of squares and the significant effects of environment in all models for gari and fufu%. Also, a significant effect of GEI was found for these traits, thereby complicating the breeders’ quest for developing a stable variety, because neither genotype nor environment effect can effectively capture all variation observed. This will require testing of genotypes in diverse environments before selection can be made. The GGE biplot enables a visual comparison of the locations and genotypes, and their interrelationships and performance potential of genotypes. The biplot (Figure 7) explained 57.81 and 61.05% of the total G + GE interactions of gari and fufu%, respectively, indicating that there is more environment and interaction contribution to performance of a genotype.

Genotypes that are above population means and stable must be considered as selection candidates simultaneously to exploit the beneficial effects of GEI and to have a more accurate selection for traits improvement. Based on genotypic performance across environment and traits, different genotypes emerged as stable performers for different traits as shown by the computed Wi and GAI (Supplementary Table 7). The genotype ranking from both stability analyses suggests that there are some generic relationships between traits which supports the correlation analysis done earlier in this study. However, Wi and GAI ranked genotypes differently as their top 5, this is not surprising as previous studies suggest that Wi and GAI are negatively correlated in measuring stability (Mohammadi and Amri, 2008; Mohammadi and Nader Mahmoodi, 2008). Notwithstanding, Wi and GAI ranked G40 as part of top5 genotypes for gari% but there were no overlapping genotypes for fufu% in the study. For plant breeding purposes, the dynamic stability measure is preferred for genotype selection and trait improvement because it assumes that all genotypes responds differently to change in environmental conditions (Changizi et al., 2014). Therefore, genotypes selected as top 5 using GAI for gari% (G56, G50, G40, G23, and G55) and fufu% (G56, G24, G21, TMEB419, and G23) are recommended.

## Conclusion

There is a measurable degree of genetic variation among genotypes for the root conversion rate for gari and fufu%, making it possible to make progress through conventional selection and advancement of clonal genotypes. However, there is a need for further optimization of the data collection process and introducing high throughput data collection methods. Dry Matter content and Peel loss% had the highest correlation and could be used as a selection proxy for gari and fufu conversion rate. However, the rate of the genetic gain obtained per year might not be the same for as recorded for Dry Matter content and needs further investigation. It would be interesting to know if dry matter and root conversion rate of gari and fufu is controlled by the same genomic region/s in the cassava genome and the influence of dry matter content on quality of processed products. Apart from genetic variation, environment and interaction had a huge role to play in gari and fufu% in this study. Environmental variance is typically the most prominent most significant component of variance in populations in natural conditions. This genotypic performance suggests that a genotype with dual-purpose for high percent gari and fufu conversion rate can be bred for using one or two of the correlated environments in addition to a contrasting environment for evaluation. We have identified genotypes that performed better than the checks used in this study for gari and fufu%.

## Data availability statement

The original contributions presented in this study are included in the article/Supplementary material, further inquiries can be directed to the corresponding author/s.

## Author contributions

CA, IR, and, PK: design and study conceptualization. CA, IR, and IK: study methodology, implementation, and manuscript drafting. CA, IR, IK, and MB: formal data curation and analysis. IR, IK, SE, MB, and EP: manuscript reviewing and editing. IR, SE, IA, SO, and EP: supervision, coordination, and fund acquisition. All authors contributed to the article and approved the submitted version.

## Supplementary material

Supplementary Figure 1 Vector views of PC2 are plotted against PC1 fufu_yield.

Supplementary Figure 2 Vector views of PC2 are plotted against PC1 Gari_yield.

Supplementary Figure 3 Vector views of PC2 are plotted against PC1 Harvest_index.

Supplementary Figure 4 Vector views of PC2 are plotted against PC1 Root size.

Supplementary Figure 5 Vector views of PC2 are plotted against PC1 Root number.

Supplementary Figure 6 Vector views of PC2 are plotted against PC1 Dry root yield.

Supplementary Figure 7 Vector views of PC2 are plotted against PC1 Fresh root yield.

Supplementary Figure 8 Vector views of PC2 are plotted against PC1 Fiber content.

Supplementary Figure 9 Vector views of PC2 are plotted against PC1 Peel loss.

Supplementary Figure 10 Vector views of PC2 are plotted against PC1 Dry matter content.
