# Supplementary figures and images for: Genetic variability and genotype by environment interaction of two major cassava processed products in multi-environments
Source: Front Plant Sci. 2022 Oct 17;13:974795. doi: 10.3389/fpls.2022.974795 (PMC9618686; doi:10.3389/fpls.2022.974795)

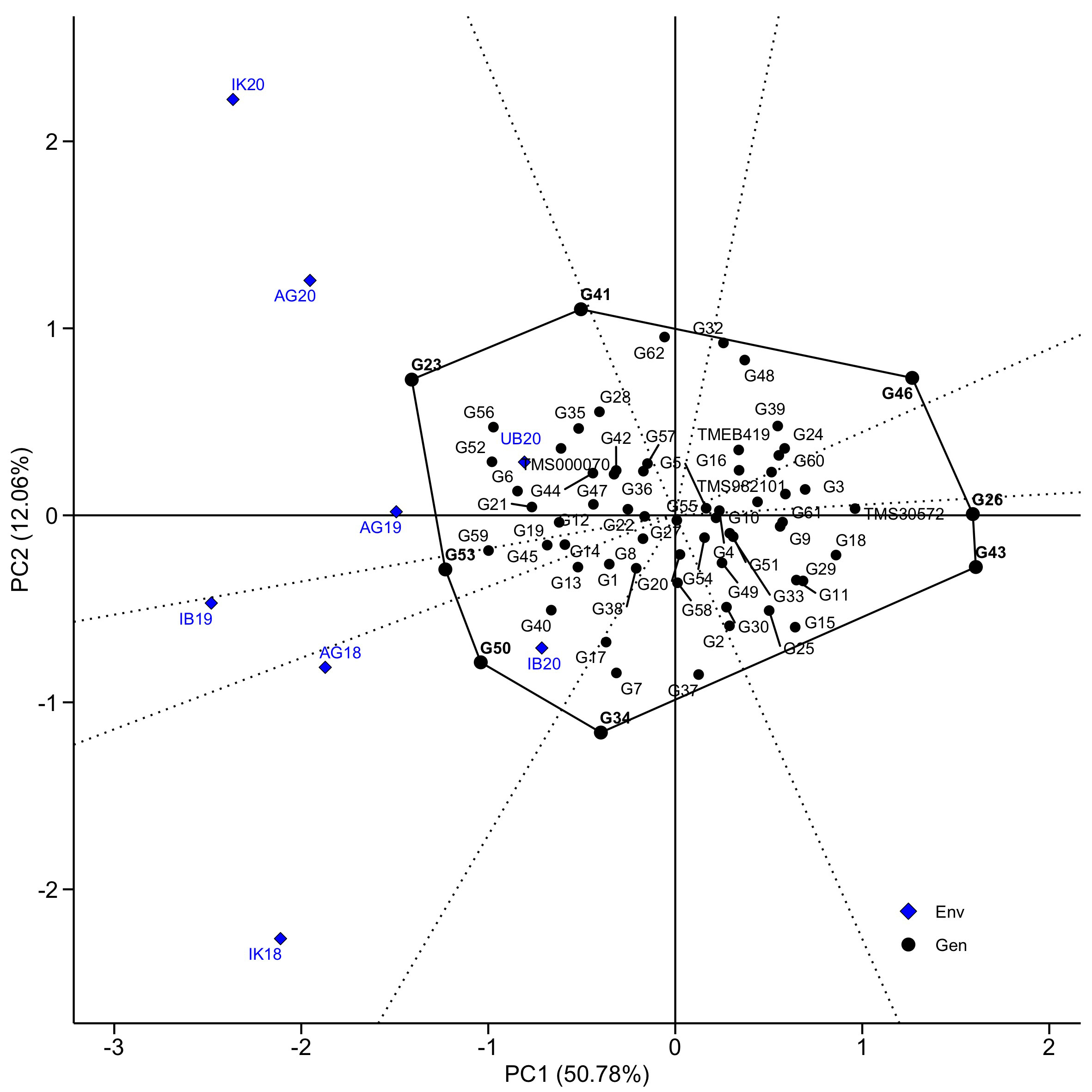

Supplement: Supplementary Figure 1 — Vector views of PC2 are plotted against PC1 fufu_yield. [file Image_1.JPEG]

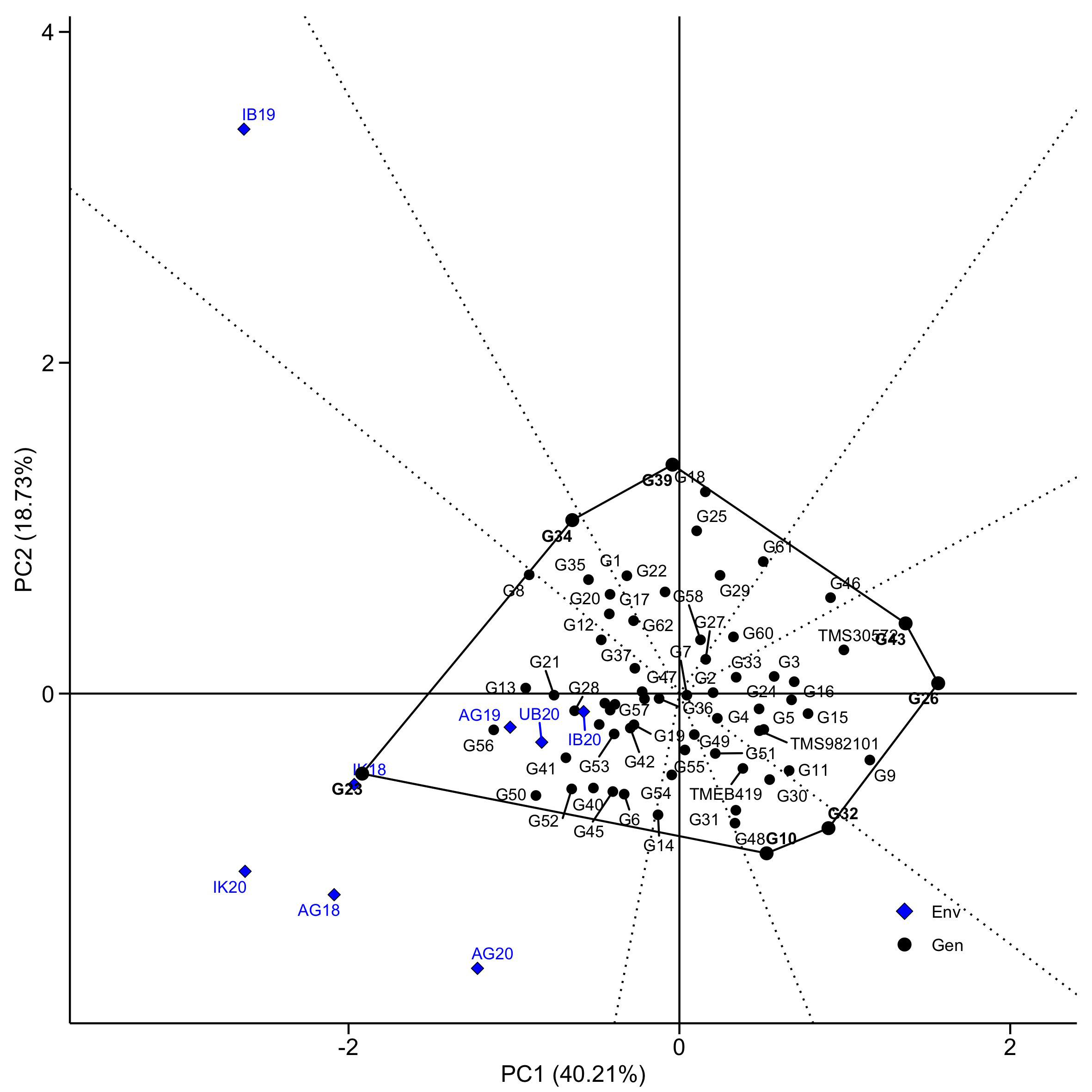

Supplement: Supplementary Figure 2 — Vector views of PC2 are plotted against PC1 Gari_yield. [file Image_2.JPEG]

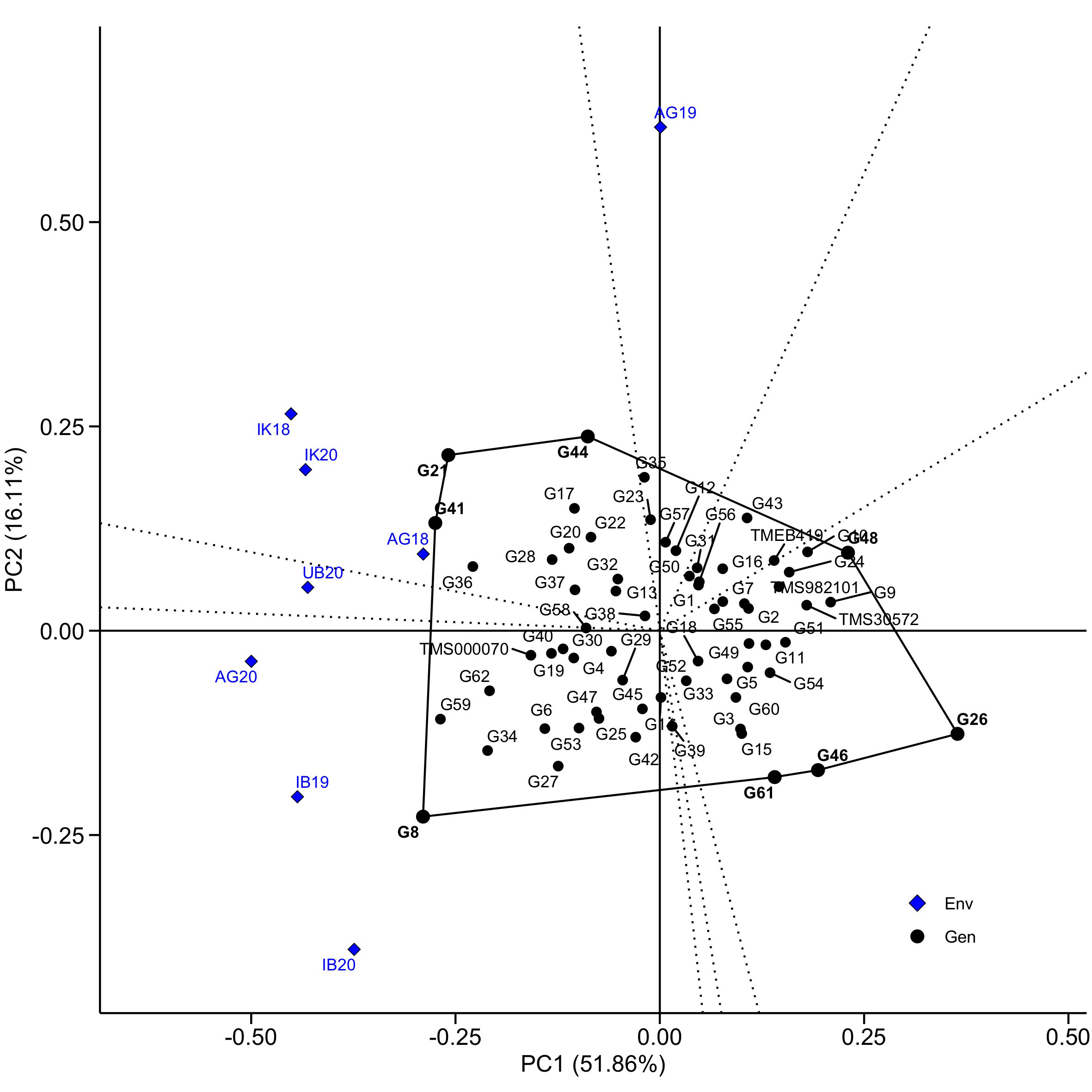

Supplement: Supplementary Figure 3 — Vector views of PC2 are plotted against PC1 Harvest_index. [file Image_3.JPEG]

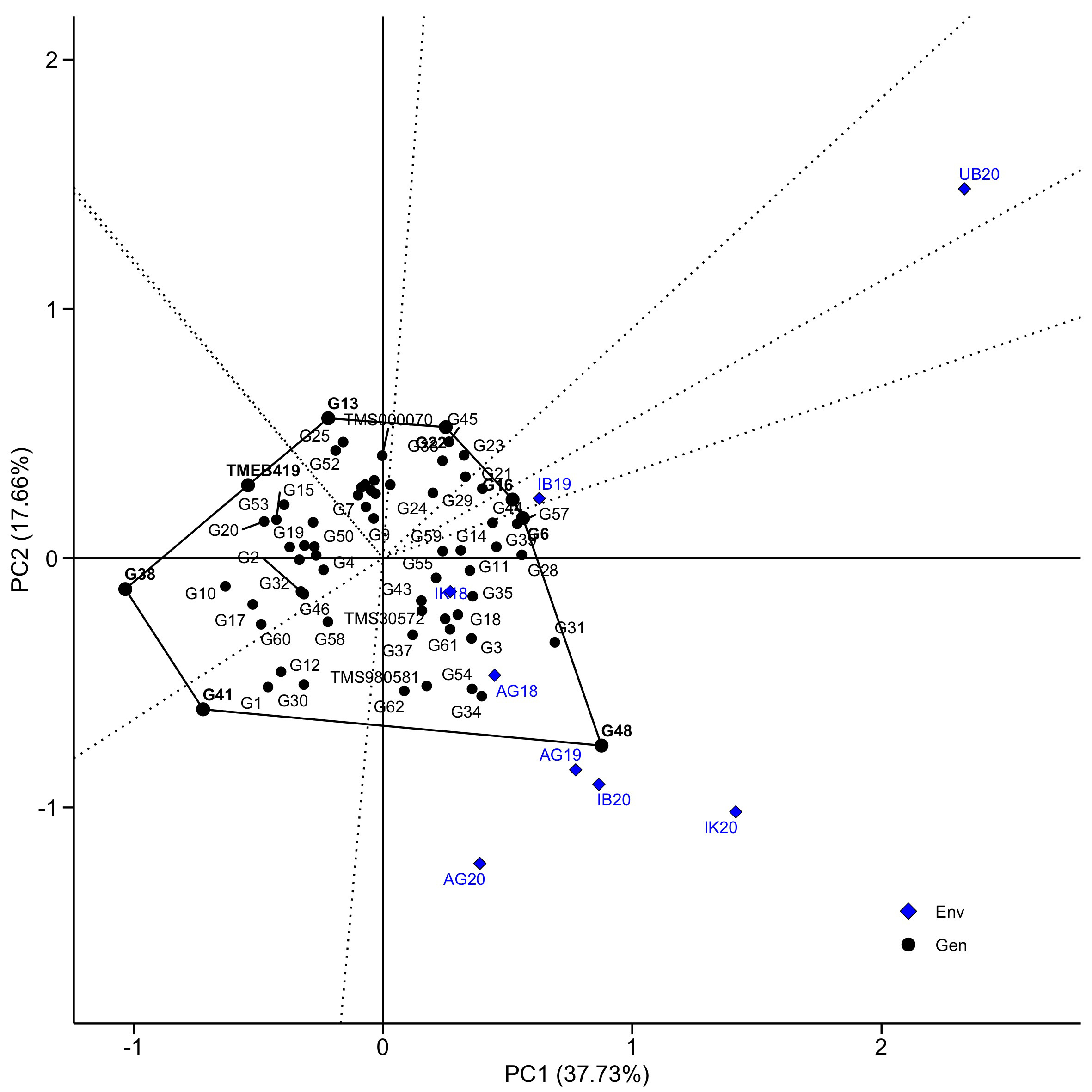

Supplement: Supplementary Figure 4 — Vector views of PC2 are plotted against PC1 Root size. [file Image_4.JPEG]

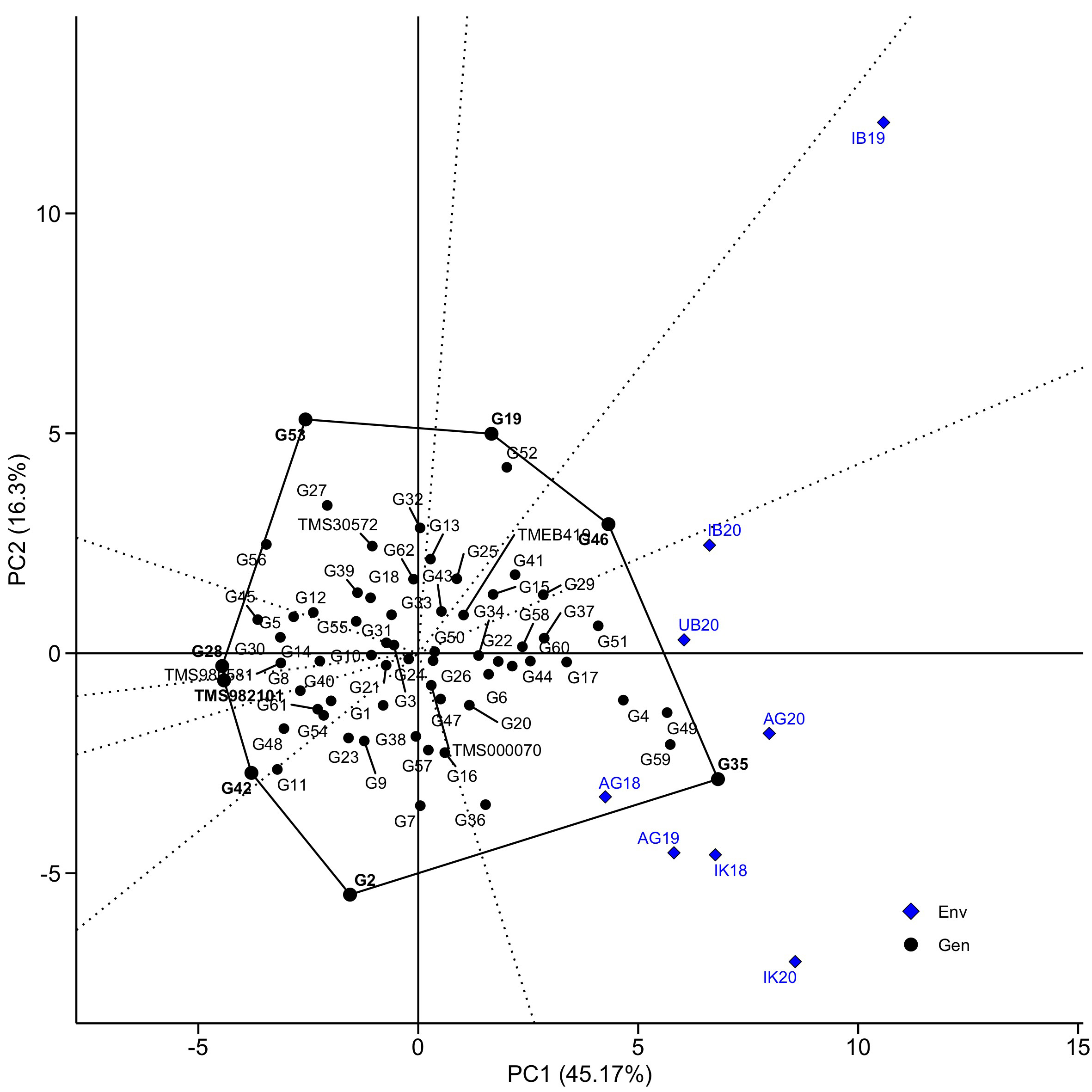

Supplement: Supplementary Figure 5 — Vector views of PC2 are plotted against PC1 Root number. [file Image_5.JPEG]

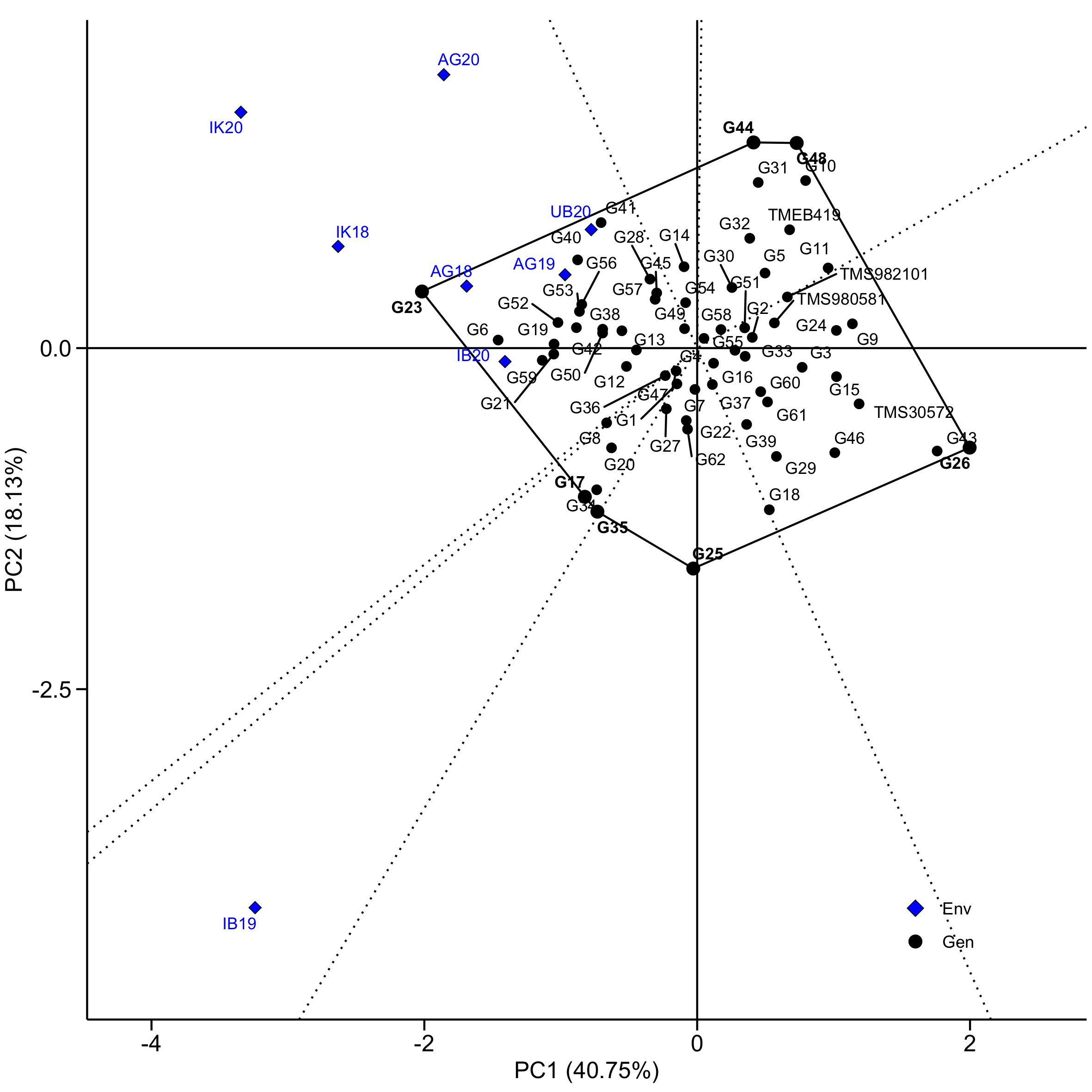

Supplement: Supplementary Figure 6 — Vector views of PC2 are plotted against PC1 Dry root yield. [file Image_6.JPEG]

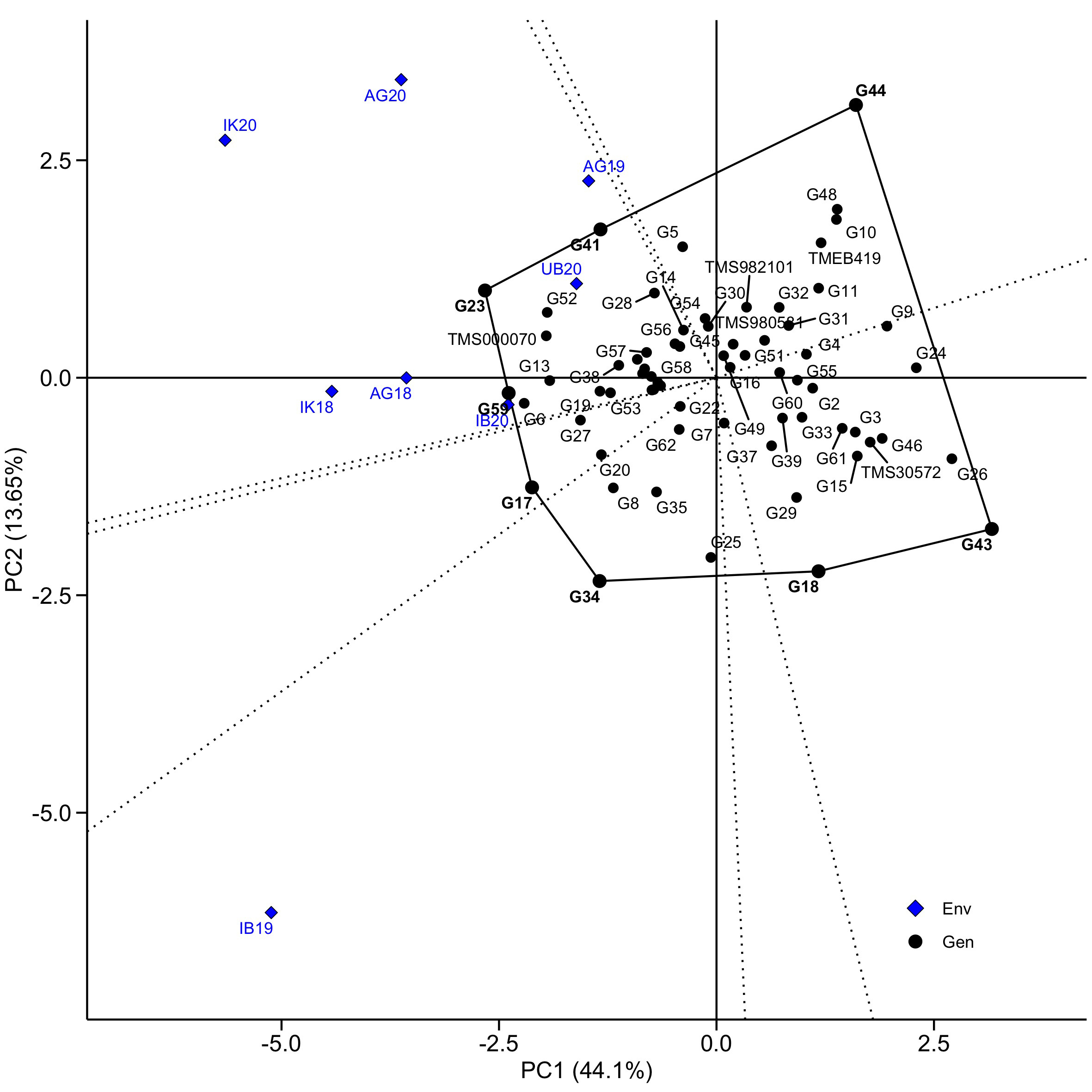

Supplement: Supplementary Figure 7 — Vector views of PC2 are plotted against PC1 Fresh root yield. [file Image_7.JPEG]

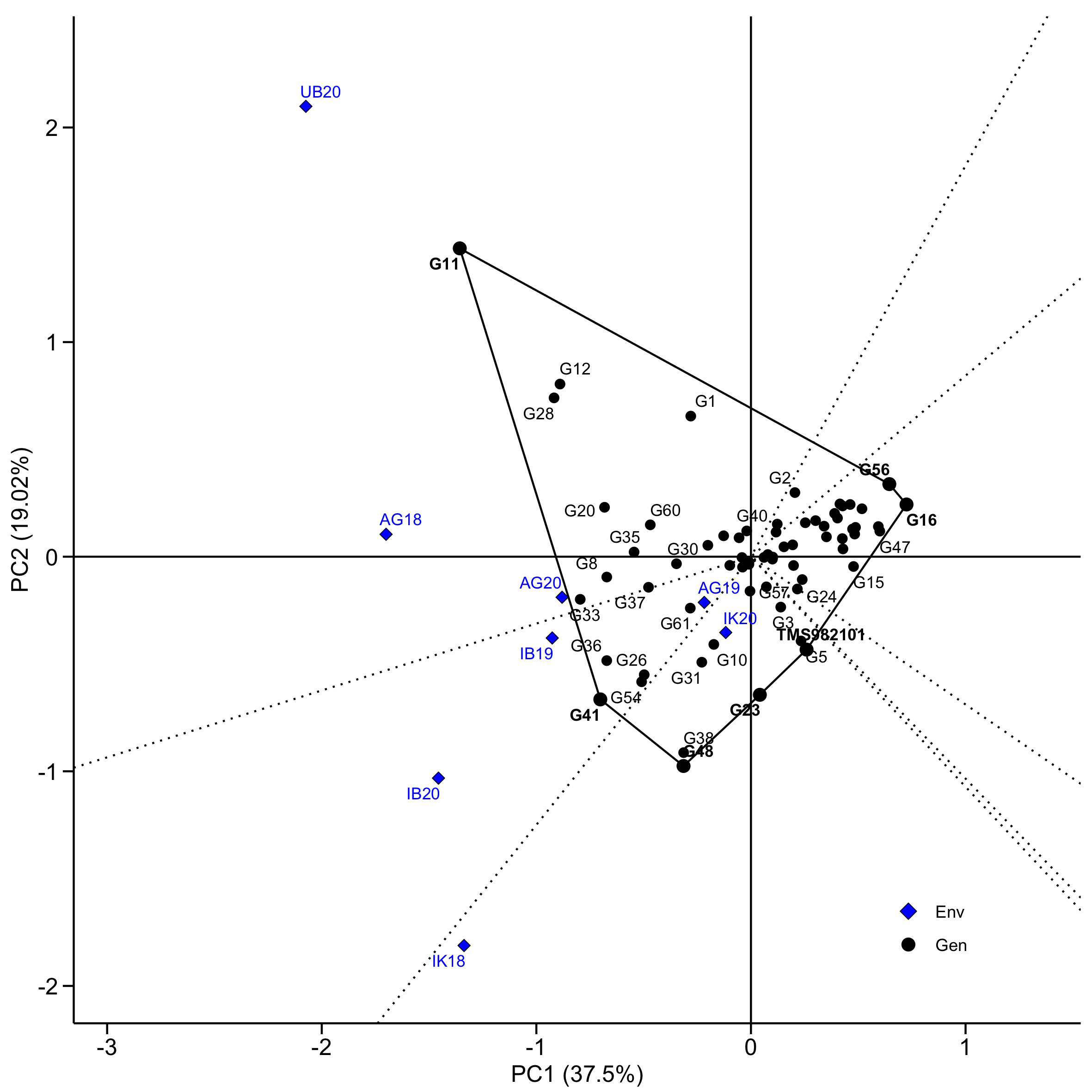

Supplement: Supplementary Figure 8 — Vector views of PC2 are plotted against PC1 Fiber content. [file Image_8.JPEG]

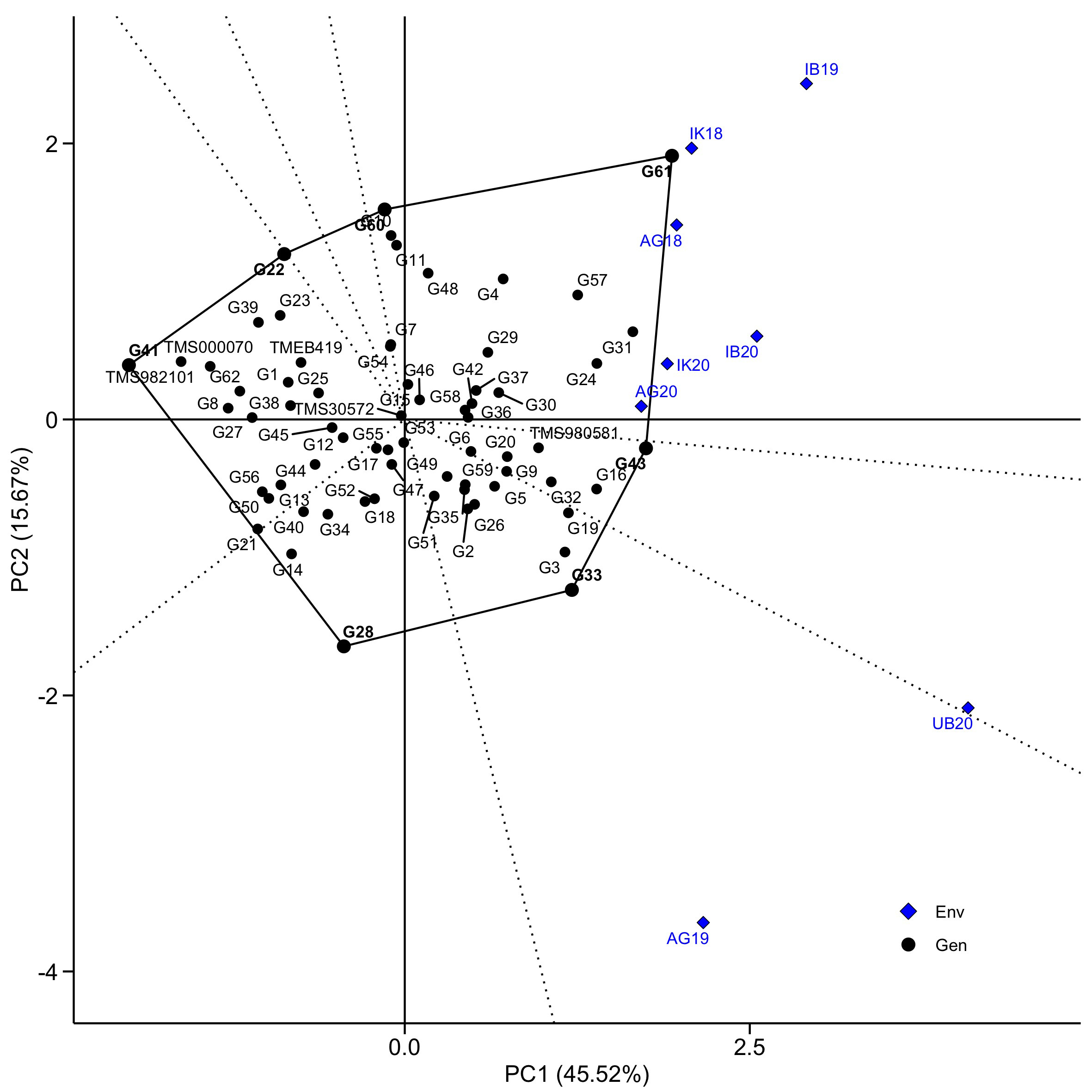

Supplement: Supplementary Figure 9 — Vector views of PC2 are plotted against PC1 Peel loss. [file Image_9.JPEG]

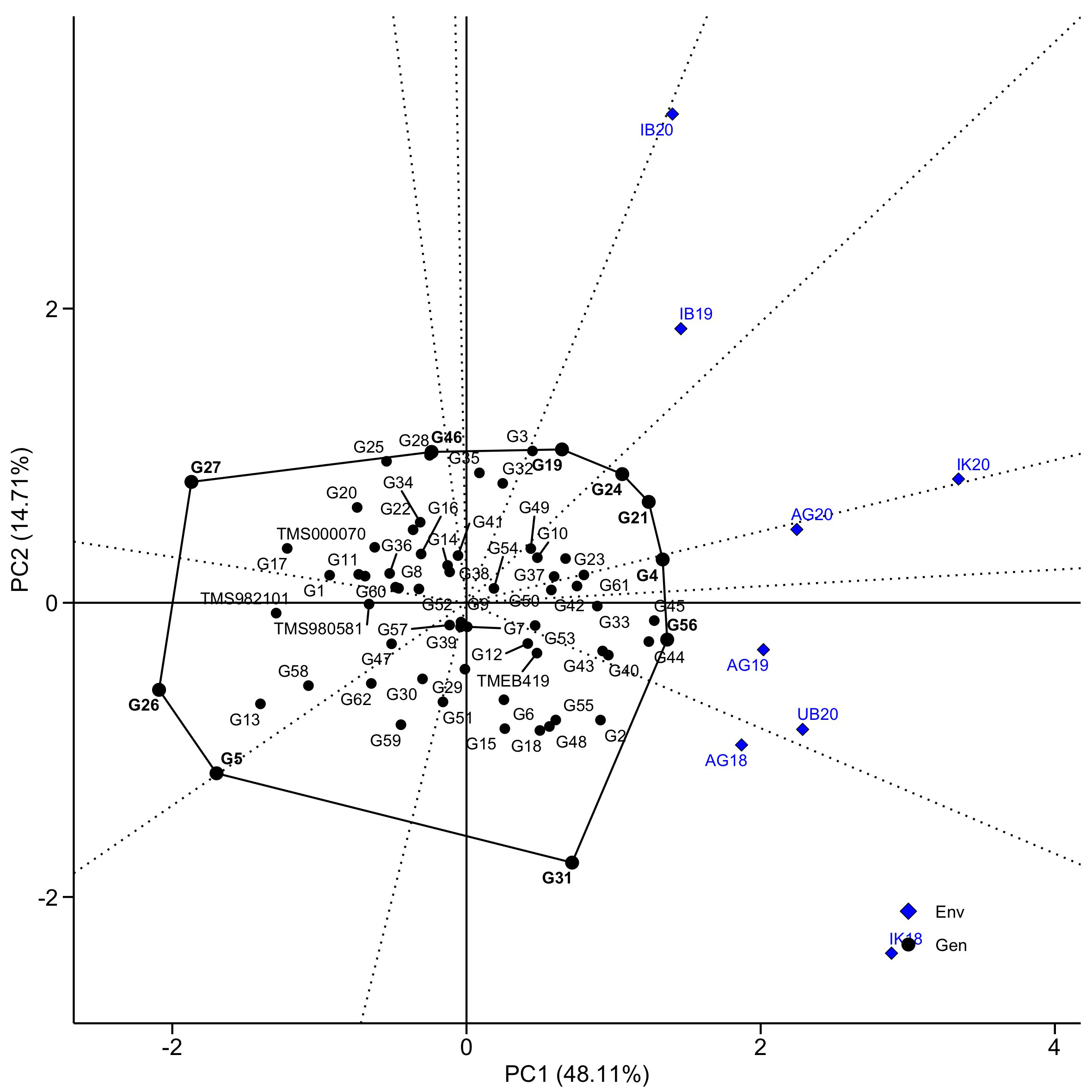

Supplement: Supplementary Figure 10 — Vector views of PC2 are plotted against PC1 Dry matter content. [file Image_10.JPEG]
